# Low Dose Prenatal Alcohol Exposure Does Not Impair Spatial Learning and Memory in Two Tests in Adult and Aged Rats

**DOI:** 10.1371/journal.pone.0101482

**Published:** 2014-06-30

**Authors:** Carlie L. Cullen, Thomas H. J. Burne, Nickolas A. Lavidis, Karen M. Moritz

**Affiliations:** 1 School of Biomedical Sciences, The University of Queensland, St. Lucia, Australia; 2 Queensland Brain Institute, The University of Queensland, St. Lucia, Australia; Virginia Commonwealth University, United States of America

## Abstract

Consumption of alcohol during pregnancy can have detrimental impacts on the developing hippocampus, which can lead to deficits in learning and memory function. Although high levels of alcohol exposure can lead to severe deficits, there is a lack of research examining the effects of low levels of exposure. This study used a rat model to determine if prenatal exposure to chronic low dose ethanol would result in deficits in learning and memory performance and if this was associated with morphological changes within the hippocampus. Sprague Dawley rats were fed a liquid diet containing 6% (vol/vol) ethanol (EtOH) or an isocaloric control diet throughout gestation. Male and Female offspring underwent behavioural testing at 8 (Adult) or 15 months (Aged) of age. Brains from these animals were collected for stereological analysis of pyramidal neuron number and dendritic morphology within the CA1 and CA3 regions of the dorsal hippocampus. Prenatal ethanol exposed animals did not differ in spatial learning or memory performance in the Morris water maze or Y maze tasks compared to Control offspring. There was no effect of prenatal ethanol exposure on pyramidal cell number or density within the dorsal hippocampus. Overall, this study indicates that chronic low dose prenatal ethanol exposure in this model does not have long term detrimental effects on pyramidal cells within the dorsal hippocampus or impair spatial learning and memory performance.

## Introduction

The consumption of alcohol during pregnancy can have detrimental neurodevelopmental outcomes for the unborn child [1], [2]. The most recognisable and severe effects of prenatal alcohol exposure include craniofacial malformations, growth deficiency, microencephaly and mental retardation characteristic of Foetal Alcohol Syndrome (FAS) [3], [4]. However, FAS is the least common effect of prenatal alcohol exposure occurring in only 5% of births to mothers who consumed alcohol during pregnancy and is typically associated with alcoholic mothers prone to heavy drinking [5], [6]. There is a large variety of poor neurodevelopmental and cognitive outcomes relating to fetal alcohol exposure that can be grouped under the umbrella term Fetal Alcohol Spectrum Disorders (FASD) [7]–[9]. While anxiety related disorders, depressive disorders, difficulties in social interaction and engagement and problems with hyperactivity and attention are among some of these poor outcomes [10]–[15], perhaps the most commonly reported problems relating to fetal alcohol exposure are those associated with learning and memory [14], [16]–[22].

Clinical studies have repeatedly demonstrated a strong association between the consumption of alcohol during gestation and deficits in learning and memory in offspring. Although some research has shown that these deficits can persist into adulthood [17], [23], investigations of learning and memory difficulties associated with prenatal alcohol exposure are most commonly conducted during schooling, where these children exhibit poor academic performance often associated with a decreased propensity for learning as well as problems with memory retention [14], [20], [21], [24].

Interestingly, some studies in animals have reported no-effects or even subtle improvements in learning and memory performance following varying doses and periods of prenatal alcohol exposure [25]–[27]. However, the majority of animal models of FASD report deficits in learning and memory performance [2], [28]–[32]. While some studies show that these impairments persist through life [33], [34] others demonstrate recovery of learning and memory capabilities in adult animals [35].

Although there is some evidence for a dose dependant relationship between the severity of these deficits and the amount of alcohol consumed [36], [37], the majority of animal research has been conducted using offspring exposed to levels of alcohol within the moderate to high range. Very few studies have investigated the impact of relatively low levels of alcohol exposure in utero on learning and memory performance in the offspring. One exception to this is a study conducted by Savage and colleagues [38], who demonstrated spatial learning deficits in 7 month old male rat offspring prenatally exposed to peak maternal blood ethanol concentrations of 30–80 mg/dl. Moreover, Savage and colleagues [38] also demonstrated decreases in evoked release of excitatory neurotransmitters within the dorsal hippocampus of female offspring.

Learning and memory function is primarily governed by the hippocampus, a region shown to be particularly vulnerable to the teratogenic effects of alcohol [39]. Exposure to moderate to high levels of alcohol during development has been shown to reduce pyramidal cell number in the CA1 and CA3 regions of the hippocampus [40]. Alcohol exposure at the moderate to high levels during gestation has also been shown to induce marked alterations in the cytoarchitecture of pyramidal cell dendrites within the hippocampus as well as impaired long term potentiation (LTP) induction in CA1 pyramidal cells [29], [41]–[43]. Although there is some evidence of reduced functionality of pyramidal cell synapses within the hippocampus following exposure to relatively small amounts of alcohol [44], to our knowledge no study has looked at the dendritic morphology of pyramidal cells in adult and aged animals following low dose prenatal alcohol exposure.

We have recently developed a model of low dose ethanol exposure [45] and shown that exposure to this low dose is sufficient to induce an anxiety-like phenotype in adult and aged rats [46]. In the current study, we utilised the same model to determine the long term impacts of chronic prenatal exposure to low dose ethanol on hippocampal-dependent learning and memory tasks, and on the morphology of pyramidal cells within the dorsal hippocampus.

## Materials and Methods

### Ethics Statement

All animal procedures were approved by the Animal Welfare Unit of The University of Queensland and follow the code of practice for the care and use of animals for scientific purposes.

### Animals and Housing

All animals were housed under temperature and humidity controlled conditions under a 12 hr light cycle (lights on at 12:00). Female nulliparous Sprague Dawley (University of Queensland Biological Resources, St. Lucia, QLD, AUS) rats were trialled on a liquid diet modelled from the commercially available Leiber DeCarli diet [47] then time mated from 8 weeks of age. Successful mating was confirmed by the presence of seminal plugs and this was marked as embryonic day (E) 1. The pregnant females were then housed singly and randomly assigned to receive a liquid diet containing 6% (vol/vol) ethanol, 15% ethanol derived calories (EDC) (n = 15), or an isocaloric control diet (n = 16) *ad libitum* for 21 hours a day for the duration of pregnancy (E1-22/23). Water was offered for the remaining 3 hours a day (09:00–12:00) and water consumption over this period of time was recorded. Each dam was offered 80 mL of fresh diet each day. Both experimental diets contained 24.4% w/v Sustagen (Hospital Formula; Mead Johnson Nutritionals, Auckland, New Zealand), 1.04% v/v sunflower oil, 25 mg selenium (Selemite B; Blackmores, Warriewood, NSW, Australia), 73% v/v reduced fat milk, 50 mM copper sulfate (Sigma Aldrich, Sydney, NSW, Australia), 199 mM ferric citrate (Sigma Aldrich) and 303 mM manganese sulfate (Sigma Aldrich). The control diet also contained 19.6% w/v corn starch. The ethanol diet (EtOH) contained 6% v/v absolute ethanol as well as a reduced amount of corn starch (11.5% w/v) to account for the additional calories provided by the ethanol. This ensured the diets were approximately isocaloric with a total of 499.2 calories (2096.6 kJ) in the control diet and 509.4 calories (2139.5 kJ) in the EtOH diet. The dams were weighed daily at the time of diet removal but were otherwise left undisturbed. At parturition the experimental diet was removed and *ad libitum* access to standard rat chow and water was then maintained.

### Offspring

As previously reported the rat pups were weighed every 3 days from postnatal day (PN) 1 to PN28 when the pups were weaned, separated by sex and housed in littermate groups with *ad libitum* access to standard rat chow and water until behavioural testing [45]. Animals were tested in one of two age groups, 8–10 months (Adult) or 15–18 months (Aged) with no more than two males or two female littermates were used at any age to minimise any effect of litter bias. The number of animals included in the analysis for individual tests are indicated in the figure legends.

### Behavioural Testing

All behavioural testing was carried out during the dark phase of the light cycle. Prior to behavioural testing animals were moved to a testing room, in their home cages, one hour after the light cycle change and left to habituate to the room for one hour. Dim lights (∼20 lux) and a small colour camera were situated above the testing arena. Extra-maze cues (i.e. vertical and horizontal bars) were visible around the room. All trials were video recorded and animal movement tracked using automated software (Ethovision ver. 5, Noldus, Netherlands).

### Y maze

Short term spatial memory performance was assessed using a Y maze task [48], [49] with a black, PVC plastic maze consisting of three identical arms (50 cm×10 cm×30 cm) randomly designated as start, familiar or novel. The task consisted of two trials, the acquisition phase and the test phase. During the acquisition phase entry into the novel arm was blocked and rats were placed into the start arm and left to explore the start and familiar arms freely for 15 minutes. After the acquisition phase rats were returned to their home cage for 30 minutes. The rats were then placed back in the maze for the test phase, in which the novel arm was unblocked, and were allowed to explore the entire maze for 5 minutes. To prevent odour cues the bottom of the maze was lined with soiled animal bedding which was mixed between each trial. Spatial memory performance was measured as the number of entries into and time spent in the novel arm, calculated as a percentage of total arm entries and total time in the maze respectively.

### Water Maze

The water maze task employed was an adaptation of the hidden escape paradigm described by Morris [50], [51]. A black platform (10 cm d×30 cm h) was placed in an off centre position inside a circular grey acrylic tank (110 cm d×50 cm h) containing warm water (24±1°C) filled to a level 1 cm above the platform. To ensure the platform was hidden the water was made opaque using a small amount of black non-toxic water based poster paint. The paradigm consisted of 4 trials per day over 4 consecutive days in which the rats were placed at one of three different start positions for each trial and given 60 seconds to locate the hidden platform. The rat was left on the platform for 10 seconds before being picked up towel dried and returned to its home cage for a 10 minute ITI. If after 60 seconds the platform was not located the rat was guided to the platform and left there for 10 seconds. The latency to locate the hidden platform was recorded for each trial as a measure of spatial learning performance. On the fifth day the rats were given a single 60 second probe trial in which the platform was removed. To assess spatial memory performance the water maze was divided into four quadrants within the tracking software (Ethovision ver. 5, Noldus, Netherlands). The quadrant in which the platform was previously situated was defined as the target quadrant and the remaining quadrants defined as opposite and adjacent. The position where the platform had been located was also outlined and the number of times this region was crossed as well as the percentage time spent in the target quadrant was analysed as a measure of spatial memory performance.

### Tissue Collection

After behavioural testing the rats were euthanized with an overdose of sodium pentobarbital (Lethabarb; 0.1 ml/kg bodyweight). Animals were then transcardially perfused with heparinised 0.2 M Phosphate Buffered Saline (PBS) and fixed with 4% paraformaldehyde (Sigma Aldrich) in PBS solution. The fixed brains were then collected and stored in 0.4% PFA for histological processing.

### Histology

Brain tissue was prepared and analysed as described previously [46]. Power calculations indicated that a minimum of five subjects per group would be required to detect a significant difference in cell number between treatment groups at 80% power. Therefore, a minimum of n = 5 brains were used for all histological analysis. In brief, for stereological investigations 50 µm coronal sections were cut from n = 6 brains per sex, per treatment from both age groups and stained using 0.1% Cresyl Violet. Unbiased stereological analysis was performed using StereoInvestigator (ver. 9, Mbf Biosciences) software. Pyramidal cell number within the dorsal hippocampus (bregma −2.28 to −3.64; [52]) was determined by the optical fractionator method using a grid size of 150×150 µm and 50×50 µm counting frames [53]. Pyramidal neurons within the *stratum pyramidale* were counted in a total of six slices with a three slice interval between. Regional hippocampal volume was determined using the Cavalieri estimator. Regional numerical cell density was calculated per 1000 µm^3^ (total # cells/volume*1000).

Investigation of dendritic morphology was performed by submerging agarose gel (4%) blocked hemispheres Golgi Cox Solution (5% Potassium dichromate, 5% Potassium chromate, 5% Mercuric chloride) for 33 days, changing the solution weekly. These Golgi impregnated brains were then coronally sectioned at 300 µm, processed as per [54] and mounted on gelatin coated slides. Apical dendrites for six pyramidal cells per animal, per region were traced using NeuroLucida (ver. 9, Mbf Biosciences) and branching morphology determined using branched structure analysis. Neurons within the dorsal hippocampus were randomly selected and assessed to determine if tracing criteria were met. Cells qualified for tracing if there was a clearly visible cell soma, a well impregnated apical dendrite and dendritic spines were visible. All spines were counted along all branches of the apical dendrite and dendritic spine density was determined by dividing the total number of spines by the total length of the apical dendrite tree.

### Statistical Analysis

All data analysis was performed using Statistical Package for the Social Sciences (ver. 17 SPSS Inc., IL, USA). Maternal data was analysed using unpaired t tests. Water maze escape trials were analysed using three-way repeated measures ANOVA with Treatment, Sex, Age and Day as Factors. All other behavioural and histological data was analysed using three-way ANOVA with Treatment, Sex and Age as factors. Post hoc analysis with a Bonferroni correction was used where significant interactions between factors were observed. The observed power for analysis of stereology and dendrite morphology data was 0.88 and 0.77 respectively.

## Results

### Maternal data

We have previously reported maternal variables pertaining to the animals included in this study [45], [46]. In brief, *ad libitum* consumption of this diet by pregnant dams results in a peak plasma ethanol concentration (PEC) of ∼0.03±0.01% (30 mg/dl) approximately 30 minutes after offering fresh diet [45]. There were no significant differences in maternal diet consumption (28.3±0.8; 30.6±0.8 ml/day), total weight gain (97.5±6; 97.4±5 g), gestation length or litter size (10±1; 11±1) between dams fed an ethanol containing diet (EtOH) and dams fed an isocaloric control diet (Control) respectively [45], [46].

### Y Maze

**Figure 1:**
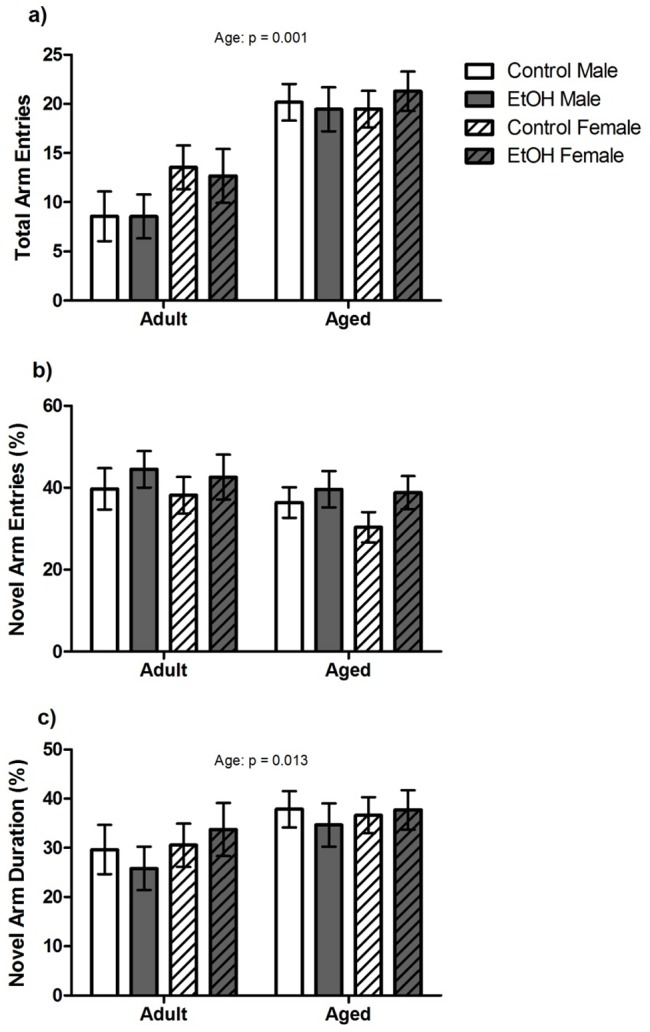
Spatial working memory performance in a Y Maze task. n = 12–15 Aged rats and n = 10 Adult animals per sex, per treatment were included in the analysis for this task. Main effects determined by 3-way ANOVA are indicated by p values above each graph. a) Aged animals made significantly more arm entries overall than Adult animals. b) There were no significant effects of age, sex or prenatal treatment on % entries into the novel arm. c) Aged animals spent a greater % time in the novel arm than Adult animals. There were no significant effects of sex or prenatal treatment on % time spent in the novel arm.

Spatial memory performance was assessed in the Y maze task by analysing novel arm exploration (Figure 1). There were no significant effects of Treatment or Sex or interactions between factors for total arm entries, percentage of entries into the novel arm or percentage of time spent in the novel arm. However, a significant main effect of Age was found for total arm entries (F(1,80) = 34.44, P<0.001) and percentage time spent in the novel arm (F(1,80)  = 6.53, P<0.05) with Aged animals making more total arm entries and spending a greater percentage of time within the novel arm than Adult animals.

### Water Maze

**Figure 2:**
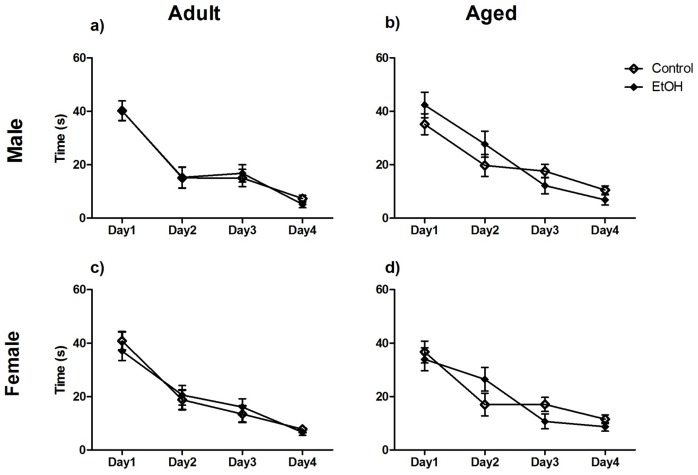
Spatial learning in the fixed platform water maze task. n = 12–15 Aged rats and n = 10 Adult animals per sex, per treatment were included in the analysis for this task. Data analysed using 3-way ANOVA but graphically separated for age and sex for visual clarity. There was a significant effect of day with all animals showing a reduction in time to escape over the four days. There were no significant effects of age, sex or prenatal treatment or interactions between these factors.

The daily average latency to escape was analysed as a measure of learning in the water maze task. This data was analysed using three-way repeated measures ANOVA but has been graphically represented separated by age and sex (Figure 2). There was a significant overall effect of Day (F(1,80) = 91.41, P<0.0001). There were no significant main effects of Treatment (F(1,80) = 0.24, P>0.05), Age (F(1,80) = 0.01, P>0.05) or Sex (F(1,80) = 0.13, P>0.05) or significant interactions between these factors.

Spatial memory was assessed using the percentage time spent in the target quadrant and number of “platform crosses” during the probe trial (Table 1). There was no main effect of Treatment (F(1,80) = 0.18, P>0.05; F(1,80) = 0.33, P>0.05), Age (F(1,80) = 0.37, P>0.05; F(1,80) = 3.71, P>0.05) or Sex (F(1,80) = 2.26, P>0.05; F(1,80) = 2.17, P>0.05) for percentage time spent in the target quadrant or number of “platform crosses” respectively. No significant interactions were found between factors for “platform crosses” but a significant Sex*Age (F(1,80) = 3.15, P<0.05) interaction was found for percentage time in the target quadrant. However, a Bonferroni post test revealed no significant differences between groups.

**Table 1:** Spatial memory performance in the water maze probe trial.

Variable	Adult (11 months)		Aged (18 months)	
	*Male*	*Female*	*Male*	*Female*
***Time in Target Quadrant (%):***				
Control	32.5±2.4	30.2±2.3	29.7±1.9	32.4±1.9
EtOH	29.6±2.4	31.5±2.4	41.5±4.7	31.9±2.0
***Platform Crosses:***				
Control	3±0.5	3±0.4	3.3±0.4	2.2±0.4
EtOH	3.1±0.5	3.5±0.5	3.0±0.6	2.4±0.4

All data presented as mean±SEM.

### Stereology

**Figure 3:**
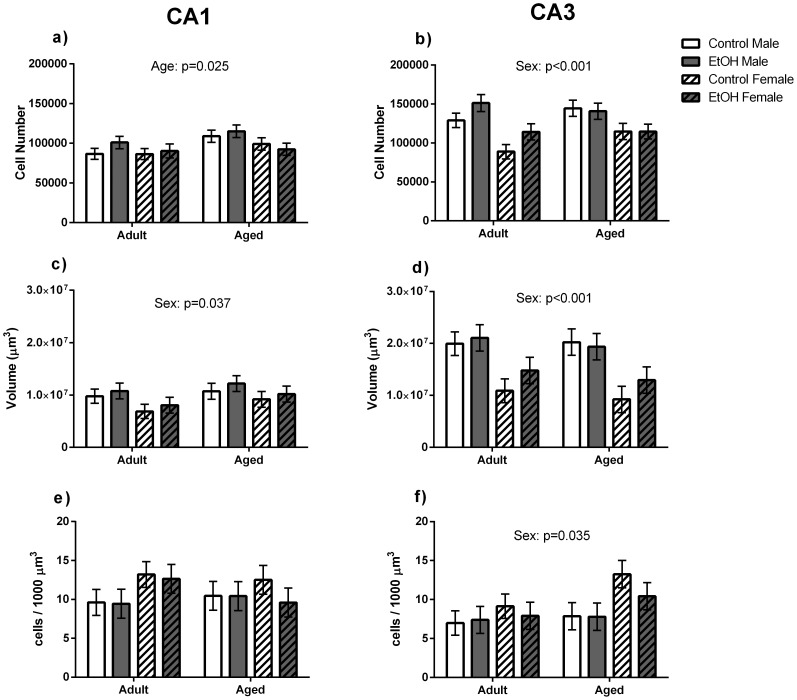
Stereological analysis of pyramidal neurons within the CA1 and CA3 regions of the dorsal hippocampus. Data from n = 6 brains per sex, age and prenatal treatment were included in the stereological analysis. a) Aged animals had a greater total number of pyramidal cells within the CA1 region than Adult animals. c) Male animals had a greater CA1 volume than Female animals. No significant differences were found for pyramidal cell density (e) within the CA1 region. b) Male animals had a greater estimated number of pyramidal neurons and a greater volume (d) of the CA3 region than Female animals. Female animals had a greater cell density within the CA3 region than Male animals.

A significant main effect of Age was found for pyramidal cell number within the CA1 region (F(1,40) = 5.618, p<0.05) where Aged animals had a greater estimated number of pyramidal cells than Adult animals (Figure 3a). A significant main effect of Sex was found for CA1 region volume (F(1,40) = 4.823, p<0.05), where male animals had greater volume of the CA1 region than female animals (Figure 3c). No other main effects of Age, Sex or Treatment or interactions between factors were found for stereological measures within the CA1 region.

A significant main effect of Sex was found for CA3 volume (F(1,40) = 21.92, p<0.01) and pyramidal cell number (F(1,40) = 21.62, p<0.01) within this region with Male animals having a greater number of pyramidal cells and CA3 volume than Female animals (Figure 3b, d). However, Female animals were found to have greater cell density (F(1,40)  = 4.96, p<0.05) than Male animals within the CA3 region. No other main effects or interactions were found for stereological measures within the CA3 region.

### Apical Dendrite Morphology

**Figure 4:**
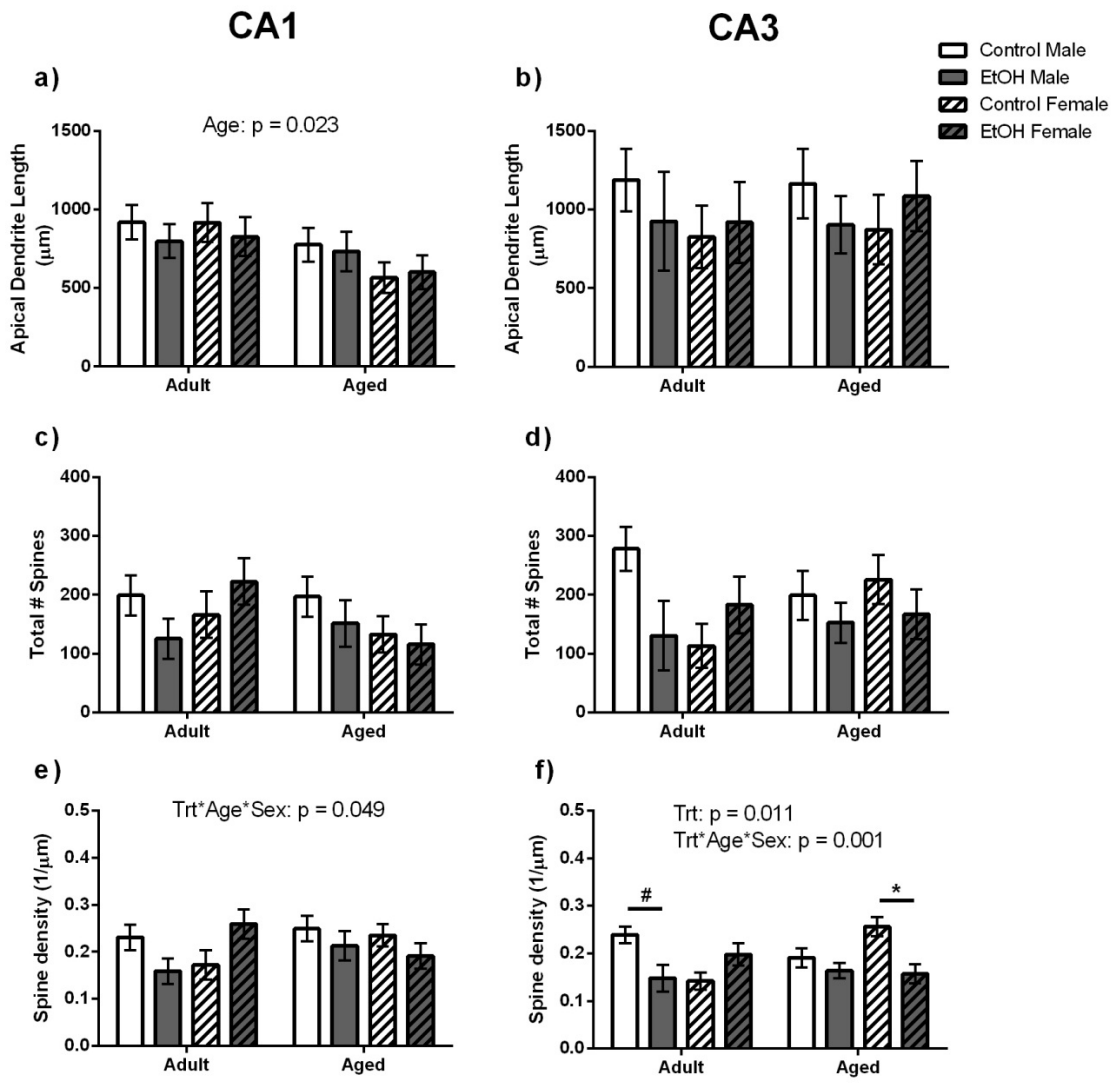
Pyramidal cell apical Dendrite Morphology within the CA1 and CA3 regions of the dorsal hippocampus. Adult animals were found to have longer apical dendrites than Aged animals within the CA1 (a) but no such difference was found within the CA3 (b). There was no effect of age, sex or prenatal treatment on the total number of dendritic spines counted within the CA1 (c) or CA3 (d) region. e) A significant interaction between Age, Sex and Treatment was found for spine density within the CA1 region. However, Bonferroni post-test analysis revealed no significant differences between groups. f) Adult Male Control animals tended to have greater spine density than their EtOH exposed counterparts (indicated by line and #) and Aged Control Female animals had significantly greater density of dendritic spines along the apical dendrite than their EtOH exposed counterparts (indicated by line and *).

A significant main effect of Age was found for apical dendrite length (F(1,40) = 5.98, p<0.05) with Aged animals having shorter apical dendrite length than Adult animals (Figure 4a). A main effect of Age was also found for the total number of branch points (F(1,40) = 12.41, p<0.01) for pyramidal cells in the CA1 region with Aged animals having less branching of pyramidal cell apical dendrites than Adult animals (5±1; 7±1 respectively). A significant interaction between Age, Sex and Treatment was also found for spine density (F(1,40) = 4.35, p = 0.049; Figure 4e). However, Bonferroni post-test analysis revealed no significant differences between groups. No other significant main effects or interactions were found for apical dendrite length, branch number, total spine number or spine density for pyramidal cell dendrites in the CA1 region.

No significant main effects or interactions were found for apical dendrite length, branch number or spine number for pyramidal cells within the CA3 region. However, a significant main effect of Treatment (F(1,40)  = 7.53, p<0.05) and an Age*Sex*Treatment interaction (F(1,40)  = 14.05, p = 0.001) were found for spine density within this region (Figure 4f). Bonferroni post-test analysis showed EtOH Adult Male animals had a strong trend towards reduced spine density than Control Adult Male animals (F(1,20) = 6.07, p = 0.057) and that Ethanol exposed (EtOH) Aged Females had a significantly reduced spine density along the apical dendrite compared to their Control counterparts (F(1,20) = 11.65, p<0.05).

## Discussion

The aim of this study was to investigate the long term consequences of low dose prenatal ethanol exposure on hippocampal-dependent spatial behaviour and morphology within the CA1 and CA3 regions of the hippocampus. Prenatal exposure to a liquid diet containing 6% (vol/vol) ethanol did not significantly impact spatial learning or memory performance in the water maze or Y maze tasks in either Adult or Aged rats of either sex. Similarly, our chronic low dose model of prenatal ethanol exposure did not induce alterations in pyramidal cell number or density in the CA1 or CA3 regions of the dorsal hippocampus. Interestingly, there was a subtle decrease in apical dendrite spine density for CA3 pyramidal cells of ethanol exposed animals that did not occur within the CA1 region. This may be indicative of a reduction in synaptic connectivity and activity within this region [45], [46], that is not sufficient to induce overt deficits in hippocampal-dependent learning and memory performance.

The Y maze is often used to investigate spatial memory function in rodent models of acute and chronic stress [48], [49]. Few studies however, have utilised the Y maze task in prenatal alcohol exposure models. The results from our study suggest that chronic low dose prenatal ethanol exposure does not affect spatial working memory in adult or aged rats. Interestingly, Dobson et al [55] utilised an adapted Y maze protocol as well as a dry land version of the Biel Maze task to assess spatial learning and memory in Guinea Pigs exposed to moderately high amounts of ethanol prenatally. They found deficits in performance in the dry land Biel Maze but, similar to our study, no deficits in performance in the Y maze task in their ethanol exposed animals. The authors conclude that the complexity of the dry land Biel Maze may be more sensitive to elucidating prenatal ethanol induced deficits in spatial learning and memory than the relatively simple two arm choice paradigm of the Y maze task [55].

In contrast to the infrequent utilisation of the Y maze task, many rodent models of FASD have employed variations of the Morris water maze paradigm to demonstrate deficits in spatial learning and memory function [28], [30], [31], [56]. Differing from the chronic low dose consumption model utilised in our study, the majority of these studies model moderate to high dose exposure to ethanol and often employ a binge type exposure paradigm. Furthermore, dose response investigations of prenatal alcohol exposure have shown a dose dependent relationship between peak maternal blood ethanol concentrations and the severity of learning and memory deficits in offspring, often finding no significant impairment at very low doses [37], [57]. However, Savage and colleagues [38] demonstrated that prenatal exposure to a liquid diet containing 3% (vol/vol) ethanol resulting in similar peak maternal blood alcohol levels as our model (∼30 mg/dl), was enough to reduce improvement in escape latency between trials in a moving platform Morris water maze task in adult male Sprague-Dawley rat offspring. Similarly, Sutherland and colleagues [58] demonstrate deficits in learning and memory performance in a moving platform Morris water maze paradigm after prenatal exposure to a 5% (vol/vol, 26% EDC) liquid diet in adult Sprague-Dawley rat offspring. It is interesting to note however, that both of these studies also show that in a fixed platform Morris water maze task, similar to the one employed in our study, these offspring did not differ from control offspring in escape latency or probe trial performance [38], [58]. This suggests that low dose prenatal ethanol exposure may not affect basic spatial learning and memory function but that more complex learning and memory processes may be impaired.

While many studies have shown regional cell loss in the hippocampus following prenatal alcohol exposure [40], [59], [60], we did not find any differences in pyramidal cell number or density in the CA1 or CA3 regions of the dorsal hippocampus in ethanol exposed offspring. However, other studies have shown no difference in cell number and density within the CA1 and CA3 regions in neonatal (PN10) and adult (PN112) rat offspring exposed to moderate to high levels of alcohol during the gestational period equivalent to the first two trimesters of human pregnancy (G1-22/23), the same exposure period used in our study [61], [62]. Also similar to our findings, Tran and Kelly [61] show greater pyramidal cell numbers in male animals than female animals. However, the gender difference in the Tran and Kelly [61] study was consistent throughout the CA1, CA3 and Dentate Gyrus, while in our study the gender difference in pyramidal cell number was only significant in the CA3 region. Furthermore, mouse models using a similar exposure period have also shown a null effect of moderate prenatal alcohol exposure and chronic alcohol exposure on hippocampal-dependent behaviour or hippocampal morphometry [63], [64].

Perhaps some of the most intriguing results from our study come from the spine density along pyramidal cell apical dendrites within the CA3 region. Previous research has demonstrated reductions in dendritic spine density as well as dendritic branching within the hippocampus, particularly within the CA1 region and the Dentate Gyrus [42], [43], [60]. We report a main effect of prenatal treatment for spine density within the CA3 region, suggesting an overall reduction in spine density for prenatal ethanol treated animals compared to control animals. This finding is lent some support by Tanaka and colleagues [41], who demonstrated a reduction in synaptic junctions within the CA3 region of near term rat pups (GD21) after maternal consumption of 5, 10 and 20% ethanol throughout gestation. However, we also report a significant interaction between prenatal treatment, age and sex for CA3 apical dendrite spine density in our animals. Previous studies have demonstrated recovery of prenatal alcohol induced synaptic deficits within the DG and CA1 region with age [42], [43]. While there was a strong trend for a reduction in spine density in adult males following prenatal ethanol, this difference was not apparent in aged male animals. At face value, this might suggest that prenatal ethanol treated male offspring exhibit a similar recovery of spine density in aged animals compared to adult animals. However, this mitigation of the difference in spine density appears to be driven by a reduction in density in control animals while, spine density in ethanol male animals appears unchanged between age groups. Similarly, the apparent gain of spine density deficits in the CA3 region for aged prenatal ethanol treated females appears to be primarily driven by an increase in spine density in control female animals between the age groups with only a slight reduction in apical dendrite spine density in prenatal ethanol treated aged females.

Within the literature, administration of ethanol through a liquid diet is usually compared to a pair-fed control group to account for potential confounders associated with malnutrition [47]. Chow fed controls have also been widely used within the literature to compare the effects of the diet or diet restriction (pair-fed), without alcohol, on the growth and development of offspring [38], [65]–[67]. As the EtOH-exposed dams in the current study showed no sign of malnutrition, consumed the same amount of diet per day as the Control dams and both diets were nutritionally complete and approximately equivalent in caloric content, we felt that the inclusion of a specific pair-fed group was unnecessary. Equally, in the current study normal growth did not appear to be altered as the dams from both treatment groups showed similar weight gain patterns to those of a chow fed group (47±3 g between E12–20) of the same rat strain, included in a previously study published by this laboratory [68].

Overall, within this study we did not find any deficits in spatial learning and memory ability or long term alterations in pyramidal cells within the CA1 and CA3 region of the dorsal hippocampus using a model of chronic low dose prenatal ethanol exposure. While the lack of deficits found within this study might seem somewhat reassuring to those who consume small amounts of alcohol during pregnancy, these findings should be taken in context. Our lab has previously reported subtle effects on growth and development in ethanol exposed offspring using this chronic low dose model [45]. Furthermore, there exists evidence of regional selectivity for ethanol-induced changes in the brain [69]–[71]. In line with this, we have recently shown that this model of chronic low dose prenatal ethanol exposure results in an increase in dendritic spine density and branching in the Basolateral (BLA), but not the medial (MeA) or central (CeA) nuclei of the amygdala in conjunction with a subtle phenotype for anxiety-like behaviour in these animals [46]. Collectively, these data suggest that consumption of small amounts of alcohol during pregnancy can lead to long term changes in offspring in some brain structures (BLA) while having no effect on others (hippocampus).
